# Achievement of Vancomycin Therapeutic Goals in Critically Ill Patients: Early Individualization May Be Beneficial

**DOI:** 10.1155/2016/1245815

**Published:** 2016-03-17

**Authors:** Bita Shahrami, Farhad Najmeddin, Sarah Mousavi, Arezoo Ahmadi, Mohammad Reza Rouini, Kourosh Sadeghi, Mojtaba Mojtahedzadeh

**Affiliations:** ^1^Clinical Pharmacy Department, Faculty of Pharmacy, Tehran University of Medical Sciences, Tehran 14155/6451, Iran; ^2^Department of Clinical Pharmacy and Pharmacy Practice, Faculty of Pharmacy and Pharmaceutical Sciences, Isfahan University of Medical Sciences, Isfahan 71746-73461, Iran; ^3^Anesthesiology and Intensive Care Department, School of Medicine, Tehran University of Medical Sciences, Tehran 14155/6451, Iran; ^4^Department of Pharmaceutics, Faculty of Pharmacy, Tehran University of Medical Sciences, Tehran 14155/6451, Iran; ^5^Research Center for Rational Use of Drugs, Tehran University of Medical Sciences, Tehran 14155/6451, Iran

## Abstract

*Objective.* The aim of our study was to assess and validate the effectiveness of early dose adjustment of vancomycin based on first dose monitoring in achieving target recommended goal in critically ill patients.* Methods.* Twenty critically ill patients with sepsis received loading dose of 25 mg/kg of vancomycin and then were randomly assigned to 2 groups. Group 1 received maximum empirical doses of vancomycin of 15 mg/kg every 8 hrs. In group 2, the doses were individualized based on serum concentrations of vancomycin. First dose nonsteady state sampling was used to calculate pharmacokinetic parameters of the patients within 24 hours.* Results.* Steady state trough serum concentrations were significantly higher in group 2 in comparison with group 1 (19.4 ± 4.4 mg/L versus 14.4 ± 4.3 mg/L) (*P* = 0.03). Steady state AUCs were significantly higher in group 2 compared with group 1 (665.9 ± 136.5 mg·hr/L versus 490.7 ± 101.1 mg·hr/L) (*P* = 0.008).* Conclusions.* With early individualized dosing regimen, significantly more patients achieved peak and trough steady state concentrations. In the context of pharmacokinetic/pharmacodynamic goal of area under the time concentration curve to minimum inhibitory concentration (AUC/MIC) ≥400 and also to obtain trough serum concentration of vancomycin of ≥15 mg/L, it is necessary to individualize doses of vancomycin in critically ill patients.

## 1. Introduction

Early optimization of antimicrobial therapy in sepsis is crucial in reducing its ominous complications, most importantly, multiple organ failure, and also in preventing bacterial resistance [1, 2]. Recent studies emphasize increasing prevalence of Gram-positive microorganisms, especially methicillin-resistant* Staphylococcus aureus* (MRSA) [3–5]. Due to the increase in prevalence of antimicrobial resistance, efforts to treat Gram-positive microorganisms led to reconsidering traditional antibiotics like vancomycin. Vancomycin is wildly used for treatment of serious Gram-positive infections involving MRSA [6]. In the absence of consensus of indications of vancomycin initiation in sepsis in the center-based guidelines, it can be used as the first line of treatment for septic patients with unknown etiology, sepsis associated with unstable hemodynamic, ventilation associated pneumonia (VAP), clinically highly suspected for soft tissues or abdominal source of infection, and catheter related infections [7–9].

Despite the vital role of vancomycin in the treatment of MRSA infections, a complete consensus has not been reached against the optimum dosing regimens and pharmacokinetic/pharmacodynamic (PK/PD) goals in critically ill patients. According to the spectrum of different patients, different levels of organ function, and physiologic alterations in critically ill patients, therapeutic drug monitoring (TDM) should be considered to achieve pharmacokinetic goals in intensive care units (ICUs) [10]. Because of altered vancomycin pharmacokinetic parameters such as volume of distribution and clearance in critically ill patients, it is recommended that the doses of vancomycin should be adjusted based on serum concentrations to achieve therapeutic levels [11, 12].

Area under the time concentration curve to minimum inhibitory concentration (AUC/MIC) and a variety of trough concentration ranges have been studied. Recent guidelines suggest that AUC/MIC ≥400 for vancomycin is the best predictor of treatment success and prevention of spread of vancomycin resistance [13, 14]. Considering that implementation of AUC/MIC in the setting of TDM requires expert clinicians to identify the optimal dose by calculating volume of distribution and clearance of the drug and to use recommended trough serum concentration of 15–20 mg/L for dose adjustment instead of AUC/MIC, American Society of Health-System Pharmacists (ASHP) therapeutic guidelines recommend that, for a pathogen with an MIC of 1 mg/L, the minimum trough serum vancomycin concentration is to be at least 15 mg/L to obtain the target AUC/MIC ≥400 [14].

Routine recommendations given by clinical guidelines for serum vancomycin monitoring in critically ill patients are based on the steady state concentrations. Actually, serum concentrations of vancomycin should be obtained no earlier than at steady state for monitoring vancomycin effectiveness. Patients must receive maximum empirical doses of vancomycin as 15–20 mg/kg every 8–12 hrs until the steady state for at least 48 hours is reached, though it could lead to underdosing in most patients even by administering a loading dose [14].

On this basis, we planned a randomized two-arm prospective study to determine the effectiveness of first-dose individualized method in comparison with routine monitoring based on steady state conditions to achieve therapeutic goals.

## 2. Methods

### 2.1. Study Design and Participants

This study was designed as a randomized clinical trial. Patients admitted to general and emergency ICU wards of “Sina” Hospital, affiliated to Tehran University of Medical Sciences (TUMS), were screened from October 2012 to December 2013 for the study eligibility during their ICU stay. In all cases, informed consent was obtained from patients or their next of kin. The study protocol was approved by the ethical committee of TUMS. Our clinical trial is registered in Iranian Registry of Clinical Trials under code number IRCT201209291497N2 and was also submitted to Research Center for Rational Use of Drugs (RCRUD) in 2012.

Inclusion in the study required that the patients be older than 18 years and have normal renal function (defined as eGFR ≥60 mL/min estimated with Cockcroft-Gault equation), with evidence of sepsis following systemic inflammatory response syndrome (SIRS), that is, body temperature less than 36°C or greater than 38°C, heart rate more than 90 beats/minute, respiratory rate more than 20 breaths/minute and/or an arterial partial pressure of carbon dioxide less than 32 mmHg, white blood cell count (WBC) of less than 4000 cells/mm^3^ or more than 12000 cells/mm^3^, survival prognosis more than 72 hours, recent onset of vancomycin administration, and no history of vancomycin sensitivity. Computer-generated random numbers were used for simple randomization of subjects.

The exclusion criteria are as follows: (I) younger than 18 years of age; (II) previous administration of vancomycin before the current infection; (III) renal replacement therapy; (IV) pregnancy. After enrollment patients in the following criteria were excluded from the study: patient who died within the first 72 hours, acute renal injury development during the study based on the criteria of RIFLE [15], adverse reaction of vancomycin, change in treatment with vancomycin during the first 72 hours, discontinuation of vancomycin during the first 72 hrs of treatment, loss of samples of the first 48 hours, and failure of individualization due to pharmacokinetic factors.

Demographic and clinical data were obtained from the medical notes of patients. These included sex, age, body weight, height, acute physiology and chronic health evaluation (APACHE) scores, and estimated glomerular filtration rate (eGFR) upon the initiation of vancomycin. Sepsis workup and SIRS criteria exploration were performed for every patient at the baseline. All relative routine critical care laboratory tests were followed and documented through daily visits.

The study was performed in two main phases. In the first phase, in order to ensure avoidance of too high serum concentrations following maximum empirical dosing of vancomycin and to find standard clinical laboratory in assaying vancomycin levels, serum concentrations of vancomycin were measured without interventions in 10-bed medical-surgical ICU patients. In the second phase, we compared maximum empirical doses of vancomycin with first-dose individualized regimens to achieve pharmacokinetic goals within the first 60 hours of treatment. Twenty patients who had indication of treatment with vancomycin following sepsis received loading dose of 25 mg/kg based on actual body weight at a rate of 1000 mg/hr and then maximum empirical doses of vancomycin of 15 mg/kg every 8 hrs were administrated for all patients. Afterwards, the patients were randomly divided into two groups and based on the grouping treatment continued. Patients in group 1 received the maximum empirical doses of vancomycin of 15 mg/kg every 8 hrs. Group 2 patients received the same dose of 15 mg/kg every 8 hrs until the pharmacokinetic parameters of the patients were obtained in not later than 24 hours and then the doses were individualized as is explained in detail in the following paragraph.

Patients were blinded to antibiotic regimen during the experiment. Prescribers were also blinded to the serum concentrations of vancomycin during the study to avoid change in their practice given nephroprotective or antimicrobial strategies.

Serum concentrations of vancomycin were taken 4 times during the study and included (I) one hour after the end of the first-dose infusion (loading dose); (II) four to six hours after the first sample; (III) one hour after the end of first daytime steady state dose (SS peak); and (IV) one hour before the next consequent dose (SS trough).

In group 1 where all patients received fixed doses of vancomycin every 8 hrs, the steady state plasma concentrations were defined as a first dose of daytime after 48 hours of loading dose. However, in group 2, the one with no fixed intervals, the steady state plasma concentrations were determined based on an individualized vancomycin half-life.

Blood samples were centrifuged at room temperature at 3000 rpm. Blood plasma was then separated and sent directly to the laboratory for quantification of vancomycin level. In all, 108 samples were analyzed within 2 hours by means of fluorescence polarization immunoassay (Siemens Healthcare Diagnosis, UK, EMIT).

### 2.2. Pharmacokinetic Calculation and Early Individualization

By measuring trough and peak serum concentrations of the first dose (loading dose) of vancomycin, pharmacokinetic parameters were calculated including elimination constant rate (*K*_el_), volume of distribution (*V*_*d*_), half-life (*T*_1/2_), clearance of vancomycin (Cl_vanco_), and AUC for each individual patient. These parameters were calculated based on pharmacokinetic equations for one compartmental pharmacokinetic model of vancomycin. Early individualization of vancomycin was performed with dose adjustment to achieve AUC = 400–600 mg·hr/L while avoiding peak serum concentration higher than 40 mg/L, within the first 24 hrs in divided doses of every 6–12 hrs/day. Calculations were performed by the clinical pharmacists but the results were not communicated to the ICU physicians.

Parameters were calculated based on DeRyke and Alexander methods [13]. The equations were used for calculation of vancomycin dosing as follows:

(1) Kel=−ln⁡C2−ln⁡C1t2−t1,T1/2=0.693Kel,Loading  dose=Vd×Cpeak ss,Cpeak non-ss=K01−e−KeltVdKele−Kelt′,Cpeak ss=K01−e−KeltVdKel1−e−Kelτe−Kelt′,Ctrough ss=Cpeak sse−Kelt′′,τ=1−Kelln⁡Ctrough−ln⁡Cpeak+t+t′,Cl=Kel×Vd,AUC0–24=Dose  administereddrug  Clearance,

where *K*_el_ is elimination rate constant; *T*_1/2_ is half-life; *C*_peak (ss)_ is peak concentration 2 hours after infusion at steady state; *t* is duration of infusion; *K*_0_ is drug infusion rate (dose/infusion time); *V*_*d*_ is volume of distribution; *t*′ is time between end of infusion and collection of blood sample; *τ* is dosing interval; *t*′′ is the difference in time between the two plasma concentrations; *C*_peak (non-ss)_ is peak concentration 2 hours after infusion at nonsteady state; Cl is drug clearance; and AUC is area under the plasma drug concentration curve.

Based on doses of 15 mg/kg every 8 hrs that are considered usual empirical doses of vancomycin in our center, we frequently observed patients with AUC less than 400 mg·hr/L. As for the pharmacokinetic goal of AUC ≥400 mg·hr/L, we estimated that less than 70% of patients may achieve AUC ≥400 mg·hr/L. Considering the study power of 80% and level of significance of 0.05, the sample size of study was estimated to be 42 patients, that is, 21 patients in each group.

### 2.3. Data Analysis

Trough and peak serum concentrations of vancomycin and AUCs were compared in two groups through three days of study. The AUC/MIC was calculated based on the assumption that MICs were 1–1.5 mg/L for all cases. Goal target of pharmacokinetic was defined as AUC/MIC ≥400. All the analyses were performed using SPSS statistical package, version 20 for Windows*™*. All variables were tested for normality of distribution by Kolmogorov-Smirnov test. Levene's test for equality of variances was used to compare the means, and the results were analyzed based on independent *t*-test. Pearson's chi square test or Fisher's exact test was used for ordinal and nominal data. Odds ratio was used when results were statistically or marginally significant. *P* value of <0.05 was considered statistically significant for all tests.

## 3. Results

**Figure 1:**
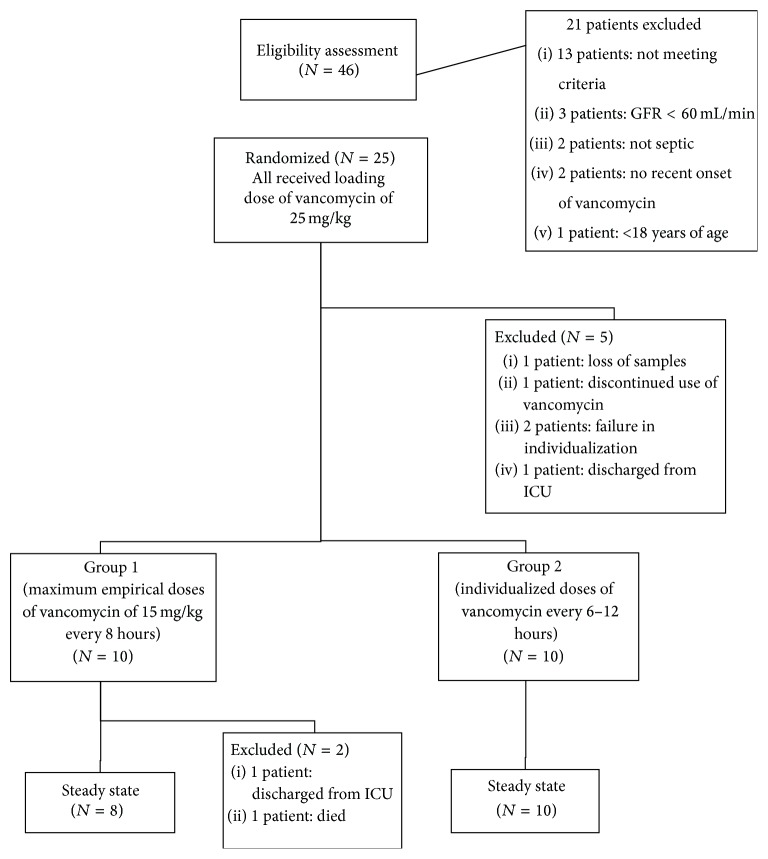
Structure of study design.

In the middle of the study, due to observation of significant differences in our primary outcome of frequencies of patients who had failed to achieve AUC ≥400 mg·hr/L in 20 patients (10 patients in each group), we halted the study to avoid wasting resources. Vancomycin serum concentrations of 108 samples were assayed. A total of 20 patients were assigned to the second phase and 2 cases were excluded based on the exclusion criteria (Figure 1).

Demographic information and pharmacokinetic parameters of the patients in the study are shown in Table 1. The differences regarding gender, age, body weight, and eGFR at the baseline in the study groups were not significant.

Steady state trough serum concentrations were significantly higher in group 2 (19.4 ± 4.4 mg/L) comparing with group 1 (14.4 ± 4.3 mg/L) (*P* = 0.03). Steady state AUCs were significantly higher in group 2 (665.9 ± 136.5) comparing with group 1 (490.7 ± 101.1), respectively (*P* = 0.008). There are no significant differences regarding average vancomycin dosage between two groups in steady state phase (44.9 ± 3.8 mg/kg versus 49.4 ± 13.1 mg/kg) (*P* = 0.33).

Table 2 pinpoints frequencies of patients who failed to achieve pharmacokinetic goals of trough serum concentrations of more than 15, 12.5, and 10 mg/L and AUCs of less than 400 and 600 mg·hr/L. Frequencies of trough serum concentrations less than 15 mg/L in steady state were significantly lower in group 2 (%10) in comparison with group 1 (%62.5; *P* = 0.04). Frequencies of AUCs less than 400 mg·hr/L in steady state were significantly lower in group 2 (%0) in comparison with group 1 (%14.3; *P* = 0.041) while frequencies of AUCs less than 600 mg·hr/L in steady state did not differ between the two groups (*P* = 0.05). With odds ratio calculation, we can say that patients who received individualized regimens are 14 and 15 times more likely to have AUC ≥600 and trough serum concentration ≥15 mg/L than patients who received fixed maximum empirical doses regimen, respectively.

Based on serum concentrations of vancomycin, 80% of patients in group 2 required individualization that indicates the need to increase or decrease the dose of 250 mg or to change dosing intervals from 8 hrs to 6 or 12 hrs.

During the study, no significant differences between the two groups were observed regarding frequencies of patients receiving vancomycin supra level with trough and peak serum concentrations of more than 25 and 40 mg/L (*P* = 1, *P* = 1).

## 4. Discussion

This study describes the value of early individualization of vancomycin in critically ill patients. To our knowledge, this is the first published study that evaluates the effects of early individualized method for achievement of pharmacokinetic goals in critically ill adult patients. With early individualized dosing regimen of vancomycin, significantly more patients achieved peak and trough steady state concentrations without additional venous sampling. The results demonstrate that an early individualized regimen of vancomycin is more likely to lead to PK/PD targets of AUC/MIC ≥400.

According to consensus review of American Society of Health-System Pharmacists (ASHP), Infectious Disease Society of America (IDSA), and Society of Infectious Diseases Pharmacists (SIDP) guidelines, empirical doses of vancomycin of 15–20 mg/kg every 8–12 hrs can be used for most patients with normal renal function to achieve trough serum concentration of 15–20 mg/L, which results in therapeutic AUC/MIC ≥400 when MIC is less than 1 mg/L [14]. However, the results of present study show that a considerable percentage of patients treated with nearly maximum empirical doses of vancomycin of 15 mg/kg every 8 hrs fail to achieve AUC/MIC ≥400, while individualized regimens of vancomycin could bring the advantage of achieving these pharmacokinetic goals. Patients receiving individualized regimens are 14- and 15-fold more likely to have AUC ≥600 and trough serum concentration ≥15 mg/L than patients receiving maximum empirical doses regimen of 15 mg/kg every 8 hrs. Hall et al. indicate that guidelines recommend that weight based empirical regimens of vancomycin are not effective in reducing mortality of patients in ICU [16]. Study by Dersch-Mills et al. also shows that empirical regimens of vancomycin fail to reach target levels in neonates [17]. Another prospective cohort study verifies that, to achieve target trough concentrations of 4 to 5 times the MIC, aggressive empirical doses of vancomycin are needed in patients with MRSA pneumonia and bacteremia [18]. Higher doses of vancomycin, that is, more than 4 g/day, are related to increased incidence of acute kidney injury (AKI) exacerbated by some factors including ICU residence [19]. Hence, therapeutic monitoring of serum concentrations would allow interventions that help reduce nephrotoxicity.

Recommendations in individual pharmacokinetic adjustments and verifications to guarantee the achievement of target serum concentrations are recently highlighted by IDSA and have led to improvements in clinical outcomes of patients [20]. Unlike the methods used in individualization of vancomycin noted in clinical guidelines that recommend measuring trough serum concentration at steady state conditions [13, 14], we found that early individualization and first-dose monitoring of vancomycin let the patients achieve therapeutic serum concentrations earlier, that is, within first 60 hrs. The results show that the first-dose monitoring of vancomycin within the first 24 hrs of initiating treatment has been effective in achieving pharmacokinetic goals in less than 60 hrs. In other words, by calculating pharmacokinetic parameters at nonsteady state conditions, there is no need to wait to reach steady state and to delay interventions for at least 60 hrs, especially in terms of MIC ≥1 mg/L. Crumby et al. performed a similar study in 108 patients and compared nomogram-based and individualized vancomycin regimens in neonates. Significantly more patients achieved peak and trough steady state concentrations after the first pharmacokinetically adjusted dose. This study also confirmed the benefits of early individualized dosing regimen without additional venous sampling [21].

While the current recommendations for vancomycin monitoring are based on using trough serum concentrations only, there is an imprecise correlation between trough serum concentration and AUC [22, 23]. By calculating individualized vancomycin AUC through two serum concentrations for each patient, we observed patients with trough serum concentration less than 15–20 mg/L, while AUC was in the target range. Pai et al. conclude that trough only concentration monitoring method of vancomycin fails to calculate actual AUC value. To optimize the delivery of vancomycin, they describe two approaches for computing AUC by using two serum concentrations, that is, Bayesian method and equation-based method [24].

Due to the fact that MICs of vancomycin against* S. aureus* were commonly 1 mg/L or less, trough serum concentrations of vancomycin of 5–10 mg/L were considered acceptable [25]. It seems that almost every patient who received maximum empirical doses of vancomycin achieved this serum concentration. However, we were unable to achieve this goal because the study was called off in its early stage. Recently, research has shown that, to increase vancomycin MICs and guideline recommendations of serum trough concentration of 15–20 mg/L, individualized regimens are preferred. Lodise et al. found that vancomycin MIC ≥1.5 mg/L was associated with a 2.4-fold increase in treatment failure of MRSA bacteremic patients when compared with individual patients with MICs of ≤1 mg/L (36.4% versus 15.4%, resp., *P* = 0.049) [26]. In addition, irrespective of MIC the failure or success did not correlate with the attainment of primary trough of at least 15 mg/L.

This study has, however, met some limitations. First, MIC was not determined in our study though reports by the European Committee on Antimicrobial Susceptibility Testing (EUCAST) between 2013 and 2016 indicate that vancomycin susceptibility breakpoints for* S. aureus* are 2 mg/mL [27, 28]. Accordingly, the therapeutic level was set to be much higher than the breakpoints. The small sample size of this study could be another limitation of this study. Due to achievement of significant differences in our primary outcome of frequencies of patients who had failed to achieve AUC ≥400 mg·hr/L, namely, 20 patients (10 patients in each group), we had to stop in the middle of the study to avoid wasting resources. Considering important pharmacokinetic changes with advancing age [29, 30], another limitation of this study could be the lack of separate assessment of vancomycin dosing regimens in elderly patients.

## 5. Conclusion

Although maximum empirical doses of vancomycin present low risk of toxicity and can be used in all septic patients with normal renal function under therapeutic drug monitoring, PK/PD targets of AUC/MIC ≥400 may not be achieved in terms of relative antimicrobial resistance. Therefore, based on the rise of the incidences of VISA and germs with higher MICs, it is necessary to individualize doses of vancomycin in critically ill patients. Furthermore, delay in pharmacokinetic parameters calculations to reach the steady state condition may put the patients at the risk of undertreatment for at least 72 hrs.

In summary, this study suggests that first-dose pharmacokinetic monitoring and early individualization of vancomycin should be considered for critically ill patients in order to achieve pharmacokinetic targets in trough serum concentrations more than 15 mg/L and AUC ≥400 mg·hr/L.

## Figures and Tables

**Table 1:** Demographic and pharmacokinetic parameters in two groups of patients.

	Measures	Group 1	Group 2	Sig. (2-tailed)
		Mean ± SD	Mean ± SD	
	Number of patients	10	10	—

Baseline	Sex (male/female)	8/2	8/2	0.61
	Age (year)	48.5 ± 18.3	47.7 ± 20.9	0.93
	Body weight (kg)	66.0 ± 12.1	73.0 ± 12.2	0.24
	Height (cm)	170.1 ± 9.1	171.5 ± 13.9	0.78
	APACHE score	10.6 ± 1.7	13.3 ± 1.4	0.31
	eGFR (mL/min)	99.8 ± 49.8	112.4 ± 45.7	0.58

	Number of patients	8	10	—

Steady state	eGFR (mL/min)	111.1 ± 57.2	113.2 ± 45.9	0.93
	Total dose (mg/kg)	44.9 ± 3.8	49.4 ± 13.1	0.33
	Trough (mg/L)	14.4 ± 4.3	19.4 ± 4.4	0.03
	Peak (mg/L)	28.1 ± 6.0	33.7 ± 6.7	0.07
	*V*_*d*_ (L/kg)	0.65 ± 0.12	0.68 ± 0.10	0.61
	Cl_vanco_ (mL/min)	45.1 ± 54.8	79.3 ± 53.7	0.20
	AUC (mg·hr/L)	490.7 ± 101.0	665.9 ± 136.5	0.01

eGFR: estimated glomerular filtration rate, AUC: area under the curve, Cl: clearance, *V*_*d*_: volume of distribution, and Vanco: vancomycin.

**Table 2:** Frequencies of patients who have subtherapeutic levels with regard to specific pharmacokinetic targets.

	Frequencies	Group 1	Group 2	Sig. (2-sided)
		%	%	
	Number of patients	8	10	—

Steady state	Trough <15 (mg/L)	62.5	10	0.043
	Trough <12.5 (mg/L)	50	0	0.023
	Trough <10 (mg/L)	12.5	0	0.444
	AUC <400 (mg·hr/L)	14.3	0	0.041
	AUC <600 (mg·hr/L)	85.7	30	0.050

AUC: area under the curve.
